# Forming Physicians: Evaluating the Opportunities and Benefits of Structured Integration of Humanities and Ethics into Medical Education

**DOI:** 10.1007/s10912-023-09812-2

**Published:** 2023-08-01

**Authors:** Cassie Eno, Nicole Piemonte, Barret Michalec, Charise Alexander Adams, Thomas Budesheim, Kaitlyn Felix, Jess Hack, Gail Jensen, Tracy Leavelle, James Smith

**Affiliations:** 1 Department of Medicine Education, Creighton University, 2500 California Plaza, Omaha, NE 68178 USA; 2 Department of Medical Humanities, Creighton University, Phoenix, AZ USA; 3 Edson College of Nursing and Health Innovation, Arizona State University, Phoenix, AZ USA; 4 Kingfisher Institute for the Liberal Arts and Professions, Creighton University, Omaha, NE USA; 5 Department of Psychological Science, Creighton University, Omaha, NE USA; 6 Creighton University, Omaha, NE USA; 7 School of Pharmacy and Health Professions, Creighton University, Omaha, NE USA; 8 Department of History, Creighton University, Omaha, NE USA

**Keywords:** Medical education, Medical humanities, Curriculum development, Curriculum revision, Identity formation

## Abstract

This paper offers a novel, qualitative approach to evaluating the outcomes of integrating humanities and ethics into a newly revised pre-clerkship medical education curriculum. The authors set out to evaluate medical students’ perceptions, learning outcomes, and growth in identity development. Led by a team of interdisciplinary scholars, this qualitative project examines multiple sources of student experience and perception data, including student essays, end-of-year surveys, and semi-structured interviews with students. Data were analyzed using deductive and inductive processes to identify key categories and recurring themes. Results suggest that students not only engaged with the curricular content and met the stated learning objectives but also acknowledged their experience in the humanities and ethics curriculum as an opportunity to reflect, expand their perceptions of medicine (and what it means to be “in” medicine), connect with their classmates, and further cultivate their personal and professional identities. Results of this qualitative study show how and in what ways the ethics and humanities curriculum motivates students past surface-level memorization of factual knowledge and encourages thoughtful analysis and evaluation about how the course material relates to and influences their thinking and how they see themselves as future doctors. The comprehensive qualitative approach reflects a holistic model for evaluating the integration of humanities and ethics into the pre-clerkship medical education curriculum. Future research should examine if this approach provides a protective factor against the demonstrated ethical erosion and empathy decrease during clinical training.

Clinical practice is inherently uncertain and reliant on experiential knowledge. Yet, high-stakes exams during pre-clerkship education, especially the USMLE Step 1 exam, primarily assess biomedical knowledge. As such, pre-clerkship curricula—including recent iterations of our curriculum— tend to emphasize the acquisition of biomedical knowledge, often at the expense of skills like listening, communication, empathy, and compassion, which are precisely the interpersonal skills that patients look for in their clinicians. While focusing on biomedical knowledge can prepare students for the tasks of diagnosing and treating biological dysfunction, it fails to prepare students for the care of patients who suffer in ways beyond their biological bodies (Piemonte and Kumagai 2019).

A 2018 Consensus Study Report from the National Academies of Sciences, Engineering, and Medicine concluded that extant research has shown “the integration of the arts and humanities with medical training is associated with outcomes such as increased empathy, resilience, and teamwork; improved visual diagnostic skills; increased tolerance for ambiguity; and increased interest in communication skills” (National Academies of Sciences, Engineering, and Medicine 2018, 3). Moreover, the report states that institutions should encourage integrative and interdisciplinary programming and “set aside resources for the hiring, research, teaching activities, and professional development of faculty who are capable of teaching integrative courses or programs” (National Academies of Sciences, Engineering, and Medicine 2018, 5). The same year this report was released, our medical education program underwent a substantial revision with a focus on organ-based systems, active learning, and early clinical experience in the pre-clerkship years. While our former curriculum included small group discussions of medical ethics cases in the first semester of the program, it did not include any medical humanities sessions, let alone intentional and longitudinal integration of the humanities. Furthermore, the former curriculum began with discipline-based courses (e.g., Molecular and Cell Biology, Anatomy, Pharmacology, and Microbiology) and had less emphasis on the integration of content between courses.

*Integration* can take on various meanings and forms depending on the academic context in which it is deployed (National Academies of Sciences, Engineering, and Medicine 2018). For instance, integration may refer to multidisciplinary faculty, including clinicians and humanists, teaching separate sessions within the same course. It may also refer to a transdisciplinary course where public health experts partner with historians to use their discipline-specific analytic approaches to create novel, population-focused strategies for improving public health education and community-oriented health strategies. In assessing options for incorporating humanities in our medical curriculum, we focused first on the structure of integration and utilized the categories of *co-curricular integration*, *within-curriculum integration*, and *in-course integration*. We also utilized faculty from separate academic disciplines, which encouraged another dimension of integration that includes such categories as *multidisciplinary integration*, *interdisciplinary integration*, and *transdisciplinary integration* (see National Academies of Sciences, Engineering, and Medicine 2018, 63–64).

Historically, our medical school had co-curricular integration of ethics supported in a single-disciplinary fashion (i.e., no integration at the faculty level). This involved ethics sessions created as a separate curriculum and presented to first-year medical students. While comprehensive in the treatment of ethics topics germane to medicinal practice (informed consent, end-of-life ethical decision-making, etc.), these ethics sessions were independently developed and presented separately from the foundational and clinical sciences courses the students experienced during the same pre-clerkship years.

Acknowledging that our goal of in-course and transdisciplinary integration may lead to a disruption of the overall medical curriculum if implemented too quickly, we proceeded with incremental changes. Our current curriculum is characterized by required credits in humanities and ethics, thus emphasizing that medical humanities is an integral, not optional, component of medical education. Some of these credits are gained through required courses, and others by courses the students may select. The humanities and ethics topics are intentionally aligned with the basic and clinical science courses in which the students are engaged at the same time, such that the topics in the contemporaneous humanities and ethics sessions match the topics of the other courses. The ethics and humanities sessions are taught or facilitated by various faculty from the School of Medicine and the College of Arts and Sciences. Thus, for clarification, we characterize our current humanities and ethics curricular status as multidisciplinary and within-curriculum integration.

Recognizing the need to incorporate the humanities into our curriculum in a way that was both meaningful to students and contributed to their personal and professional development, administrative leadership assembled a multidisciplinary group of medical school faculty, which included a scholar in the medical humanities, to develop a longitudinal humanities curriculum that incorporated active learning, small group discussion, and reflection. As part of the design process, the multidisciplinary group reviewed literature and practices at other institutions. While the curriculum was well-received by students, we were interested in developing a robust evaluation strategy to better understand the outcomes of the curricular efforts. Of particular interest was what effect—if any—the curriculum had on students’ personal and professional development, a prominent goal in many medical humanities programs (Adams et al. 2023). Many of those who have incorporated the humanities into their medical education program recognize the difficulty in evaluating such curricular efforts since measuring or assessing outcomes beyond learner satisfaction can be challenging. Moreover, while assessing the effects of individual medical humanities courses on students’ personal and professional development has been demonstrated (e.g., Aluri et al. 2023; Pitcher et al. 2022), examining the effects of a broadly integrated medical humanities curriculum is more difficult. After completing the first year of our revised curriculum, we convened a research team who was up for the challenge. Using a novel qualitative approach, we examined multiple sources of student experience and perception data, including student essays, end-of-year surveys, and semi-structured interviews with students to evaluate the outcomes of our integrated humanities and ethics curriculum.

## Background

The National Academies report states that there is a great need to “employ multiple forms of inquiry and evaluation when assessing courses and programs that integrate the humanities, arts, and STEMM fields, including qualitative, quantitative, narrative, expert opinion, and portfolio-based evidence” (National Academies of Sciences, Engineering, and Medicine 2018, 6). Following the National Academies report, the Association of American Medical Colleges (AAMC) commissioned a scoping review of the extant literature related to arts- and humanities-based programs in medical education. This review found vast, rich, and diverse literature on humanities in medical education but also identified a need for more rigorous outcome evaluation (Moniz et al. 2021). The AAMC’s 2020 report, *The Fundamental Role of the Arts and Humanities in Medical Education* (FRAHME), emphasized that research and evaluation of arts- and humanities-based programs should examine “learner outcomes beyond satisfaction with the course or program” (Howley et al. 2020, 29). Moreover, the report encouraged medical education programs to *integrate* humanities- and arts-related initiatives into curriculum design and to include more diverse voices in the outcome research, such as those of patients, learners, and humanities scholars.

Despite the fact that nearly 95% of medical schools report having required or elective courses in the medical humanities (Howley et al. 2020), there are no clear standards for how (or whether) the medical humanities ought to be incorporated into medical school curricula. As Bleakley and Marshall (2013) noted, the inclusion of such courses into the medical curriculum can be haphazard or weak. Importantly, there are also no clear standards for how students should be assessed in medical humanities or how medical humanities programs should be evaluated (Berry et al. 2023; Dennhardt et al. 2016). The tools utilized to assess cognitive ability and technical skills in medical education, such as multiple-choice tests and observed clinical performance, do not capture the essence of medical humanities or their contributions to the formation of physicians or the practice of medicine. Thus, medical educators struggle with defining and measuring the true value of medical humanities (Fins et al. 2013). In the absence of well-established approaches, evaluation of humanities programs often utilizes post-activities student surveys as the primary means of evaluation (see, e.g., Lawrence et al. 2020). Additionally, evidence suggests medical students may not even desire assessment in their medical humanities coursework (Petrou et al. 2021).

As the practice of contemporary medicine is oriented to evidence and outcomes, we ought to hold medical education to a similar standard. However, the literature on the proper role of medical humanities in medical education is significantly skewed toward descriptions of programmatic features of medical humanities in schools and defending or challenging the integration of medical humanities into the medical school curriculum rather than evidence of long-term outcomes or impact in medical practice (Ousager and Johannessen 2010). Complicating the absence of standard assessments are the multitude of research approaches that have explored the impact of medical humanities and reported on disparate sets of outcomes (Wald et al. 2019).

Perhaps due to a lack of clarity or supporting empirical evidence, many medical education programs incorporate—but fail to integrate—limited, sporadic, or elective-only humanities content into an existing curriculum. This puts humanities at odds with the prevailing science-focused paradigm of medical training, ultimately sending students mixed messages about what is *actually* important in their training (Assing Hvidt et al. 2022). In response to the calls to (a) integrate humanities initiatives longitudinally into the curriculum, (b) collaborate with faculty from both clinical medicine and the humanities, and (c) more rigorously evaluate the outcomes of such initiatives beyond student satisfaction, we describe the development of our curricular initiatives below with a specific focus on the evaluation method as a potential model for evaluating the integration of humanities and ethics into pre-clerkship medical education curriculum.

## Implementing a revised medical curriculum

Creighton School of Medicine (CUSOM)—housed within a Jesuit university in Omaha, Nebraska—implemented a revised “New ERA” (Experience, Reflection, Action) curriculum with the incoming first-year class in the Fall of 2019. While intentionally designed around organ systems, the revised curriculum also emphasizes the development of clinical skills, team-based learning, personal and professional development, and intentional reflection. Experiential learning opportunities designed to enhance the development of critical thinking and clinical reasoning skills include large-group active-learning sessions, small-group case-based learning, and clinical experiences in hospital, simulation, and community environments.

Three tracks serve as the curriculum architecture for each organ system block and span all four years of training: the “Blue Track” is comprised of the basic sciences, the “Green Track” emphasizes clinical skills, and the “Gold Track” focuses on the social contexts of care and the formation of the future physician as a professional and as a *person.* Developed through interdisciplinary faculty collaboration, the Gold Track—“longitudinal, within-program integration” of humanities and ethics (Howley et al. 2020, 9)—includes required large and small group sessions in the medical humanities, clinical ethics, leadership, personal and professional development, evidence-based medicine, patient safety, and service learning. Each week of the curriculum includes dedicated time for each of the three tracks, and although the sessions are separated by track, they cover complementary content related to the organ system. For example, during the cardiovascular course, students practice cardiovascular exams and heart-sounds interpretation during the Green Track, and they have a Gold Track session related to end-of-life care decisions and discussions (cure vs. care; humanities) and complete a case focused on the decision to turn off an automated implantable cardioverter defibrillator in a patient dying of heart failure (ethics). In addition to required Gold Track sessions, students complete at least three “Gold Selective” courses before matriculating to clerkships. Gold Selectives, created and taught by both clinical faculty and humanities scholars, are intended to enhance a deeper appreciation for medicine in the broader contexts and dimensions of society and require rigorous assessment of student learning through writing and reflection exercises. Program learning objectives define the expected learning of the program across the integrated tracks; the program has not adopted competency-based outcomes.

## Overview of the humanities and ethics curriculum

In Year 1, students participate in eight required humanities sessions and seven ethics sessions, followed by five humanities sessions and four ethics sessions in Year 2. Students complete pre-work (e.g., readings, videos, podcasts, etc.) and a 150-word written reflection before each session. Sessions begin in a large group setting where the instructor—who is a humanities scholar—sets the stage for small group discussions and shares additional content (e.g., TED Talk, short documentary) before students break into small groups with faculty facilitators.

The humanities topics, vetted by clinical faculty and humanities scholars, were selected and designed with two major learning outcomes in mind: (1) to help students approach an understanding of the lived experience of illness and suffering, including the sociocultural components of illness and health, and (2) to develop a *critical* understanding of medicine and medical education in the context of the healthcare policies and systems in which they exist. The ethics topics were selected and designed with two major learning outcomes in mind: (1) to help students develop (and reflect upon) their own ethical reasoning process and to familiarize students with the Catholic Ethical and Religious Directives (ERDs) and (2) to see the ERDs as one approach among many when thinking through clinical ethical dilemmas (see Appendix 1 for specific humanities and ethics topics).

By design, students complete at least one five-week Gold Track Selective called a “Student Interest Selectives” (SIS) during Years 1 and 2. Most SIS courses are collaboratively taught by clinical and humanities faculty with primary or secondary appointments in the School of Medicine. While courses are related to health, illness, and healthcare, they engage students in disciplines well outside the scope of traditional medical education and are intended to deepen, broaden, or even challenge students’ notions about what it means to care for others (see Appendix 1 for example topics).

In the required humanities and ethics sessions, students receive written feedback from faculty on their required session reflections throughout the year. Additionally, multiple-choice questions covering the humanities and ethic content are included on course quizzes and exams during the course blocks in which sessions occur. The summative assessment is an end-of-year student essay. These assessments are reflected in the grading for the organ system block during which they occur, and the essays are included the assessment of programmatic objectives related to personal and professional development. In the SIS, students receive feedback and are assessed on weekly or summative responses to writing prompts, which are reflected in their grades. The end-of-year summative essay was one component of the program evaluation; additional measures not specifically related to the ethics and humanities curriculum were added to evaluate the outcomes beyond the scope of the learning objectives.

## Evaluation approach

Consistent with evidence for the lack of a standard approach to evaluation (Dennhardt et al. 2016; Fins et al. 2013; Ousager and Johannessen 2010), the FRAHME report identified a need to develop rigorous evaluation methods to “measure the effectiveness of integrative models on students’ learning and workforce readiness” (Howley et al. 2020, 15). Thus, we assembled an interdisciplinary team to create a program evaluation strategy integrating multiple measures (student essays, surveys, and interviews) to evaluate the effectiveness of our longitudinal, within-program integration of humanities and ethics. Consistent with the Kirkpatrick model of program evaluation (Kirkpatrick 1959), inquiries focused on: (a) students’ perception of the Gold Track (Reaction); (b) students’ attainment of stated ethics and humanities learning objectives (Learning); (c) students’ attainment of additional outcomes not specifically stated in the learning objectives (Learning); and (d) students’ professional identity development as related to their behavioral intentions as future physicians (Behavior). The program evaluation utilized a novel, comprehensive qualitative approach that could be used as a model for holistic curricular evaluation of the integration of humanities and ethics into the pre-clerkship medical education curriculum.

## Methods

### Program evaluation

**Fig. 1:**
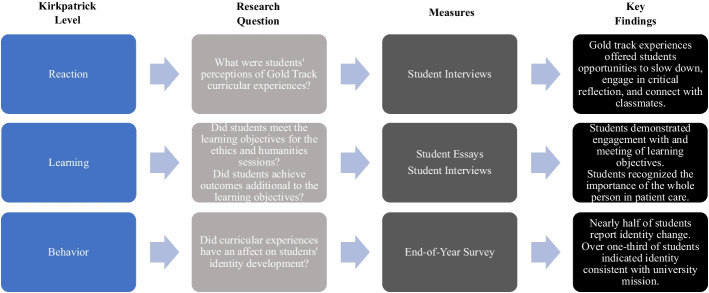
Research questions, measures, and key findings

The program evaluation included multiple measures to assess 168 (50% female, 50% male; 66% White, 17% Asian, 4% Other, 13% Not Reported) first-year students’ perception, learning, and behavioral intentions following their experiences in the new curriculum (see Fig. 1). Participation rates varied by measure and are reported below. Data collection ranged from the spring of the M1 year through the fall of the M2 year. These measures represent a subset of the school’s larger ongoing curriculum evaluation plan for the new curriculum, which also includes the collection of traditional course evaluations and quantitative measures, including the Jefferson Empathy Scale (Hojat 2016), the Intellectual Humility Scale (Leary et al. 2017), and the Social Desirability Scale (Reynolds 1982).

Some members of the research team were involved in the curricular design of the Gold Track (CE, NP, JS), and due to close proximity, it was determined they would not participate in interviews or thematic coding of essays or interviews. The research team was intentionally built to include members external to the School of Medicine (CA, TB, GJ, TL, JH) and the institution (BM, KF) to provide multiple lenses to the data. Although all authors have an inherent interest in the subject matter of the research, decisions were made with an interest in reducing our personal biases.

### Data collection and analysis

#### Interviews with students

To assess students’ perceptions of and learning in the new curriculum, in-depth, semi-structured, voluntary interviews were conducted with 27 students (63% female, 37% male; 63% White, 26% Asian, 11% Other). Students indicated their willingness to participate via a related survey of M1 and M2 students (total number of students = 332; survey results not reported here). Out of the 81 survey respondents, 52 students indicated interest and were contacted via their school-based email accounts and provided an information sheet describing the study and a schedule of specific interview dates and times. Twenty-five students declined to be interviewed or did not respond to the request. Students who participated received a $15.00 gift card.

Interviews were conducted by members of the research team (JH, CA, TL, TB) over Zoom and recorded with the participants’ permission. Recordings were automatically transcribed by the Zoom software, and transcripts were reviewed for accuracy by members of the research team. Interviewers began each session by introducing themselves, outlining the study, confirming that the participant had read and understood the consent form, and asking if there were any questions before they began the interview. Interviews lasted between 30–45 min. Utilizing a semi-structured approach, participants were asked specific pre-planned questions about their experiences with the new curriculum. Germane topics outside the prepared questions were discussed if they arose during the interview.

Interview data were analyzed using a multistep coding process (Miles et al. 2020; Saldana 2016). First, team members (BM, JH, KF) read all interview transcripts to identify common, reoccurring points, perspectives, and insights embedded within participants’ responses. This inductive, open-coding process highlighted factors such as *discussions; open-mindedness; “newness” of topics; the role of facilitator(s); voice*, *(different) perceptions*, and *other-than-curriculum*; among others.

The factors identified in the initial stage were then used as codes themselves in tandem with the prominent concepts and attributes associated with humanities and ethics-based medical curricula noted within previous literature (e.g., *humanism*, *empathy*, *other-orientation*, *gratitude*, and *context*), and transcripts were then re-analyzed to more fully explore the nature and interconnectedness of these factors, as well as reoccurring themes within the data. Although interview transcripts were analyzed independently, the coding team met throughout the analysis process to compare/contrast findings and confirm intercoder reliability. The analysis team consistently presented similar findings, and rare variations between team members were settled through discussion and consensus.

Through these analysis processes, we identified the general theme of *Opportunity* and six independent yet interrelated sub-themes related to *Opportunity*. The analysis team also identified the broad category of *Structure & Format*, with four related sub-categories, that had a significant effect on students’ experiences with and perceptions of the program.

Regarding qualitative data analysis, a category is an explicit term or concept found in the data within which other specific factors, topics, and concepts are organized—in other words, it is a particular “bucket” for related “things.” A theme, however, is a broad label used to group a particular type of finding that is not as tangible within the data. As Rossman and Rallis state, “Think of a category as a word or phrase describing some segment of your data that is explicit, whereas a theme is a phrase or sentence describing more subtle and tacit processes (Rossman and Rallis 2003, 282).” Each theme, subtheme, category, and subcategory was then used as a deductive code, and “clean” versions of the interview data were again analyzed to identify any lingering/remaining related data. “Saturation” was achieved once no evidence of new codes or themes was identified. The findings are discussed below in detail.

#### Student essays

At the conclusion of the M1 year, students completed a required essay reflecting on various content from the humanities and ethics sessions. They were asked to apply these concepts, constructs, and perspectives to their own professional development and their thoughts on what it means to be a doctor. Students were instructed to answer one of two prompts: (a) “Explore what kind of knowledge, skills, and attitudes/dispositions are needed—beyond a scientific understanding of the body—in order to take care of patients with serious illness or injury”; or (b) “Describe how you think your ability to do good clinical ethical reasoning has improved—or not improved—using your pre- and post-sessions reflections over the past year as evidence of your improvement, or lack thereof.” Students were encouraged to utilize assigned readings and other session material to support their thoughts and perspectives. For this specific study, a subset of these essays (two essays were randomly selected from each small group; *N* = 42) was analyzed to examine if, and to what extent, students engaged with the learning objectives of the humanities and ethics sessions. Since essays were randomly selected, no demographic information was reported.

Essays were analyzed utilizing a strict deductive process. Team members (BM, JH, KF) reviewed the learning outcomes for the humanities and ethics sessions and extracted specific words and phrases to use as deductive codes to analyze the essays (e.g., *illness & suffering*; *sociocultural components of illness & health*; *medicine & medical education in the context of healthcare policy and systems*; *own ethical reasoning process(es)*; *Catholic Ethical & Religions Directives (ERDs)*; *clinical ethical dilemmas*; among others). These codes were used to explore and identify direct evidence of learning objectives nested within the students’ essays. Analyses of the student essays were concluded when the coding processes identified no new results.

#### End-of-year survey

All students were required to complete an end-of-year survey reflecting on their curricular experiences as part of the regular evaluation of the medical curriculum. No demographic information is collected as part of this survey. One hundred students responded to the following prompt, designed to examine their identity development during their M1 year: “Describe your identity as a future physician. How has your identity as a physician changed during your time in medical school?” The responses were analyzed to examine if students’ identities had changed and how they conceptualized their identity as physicians. Since this survey was completed as part of the regular program evaluation process, these responses were not prompted by the context of the ethics and humanities curriculum.

Team members (CE, TB) read the full survey responses to identify common, recurrent language, perspectives, and insights embedded within the responses. Common themes in the responses included the degree of (or lack thereof) identity development and the focus of students’ descriptions of their identity. The researchers developed a codebook by which to categorize responses (see Table 2). Following that discussion, each member of the team reread the response and assigned codes independently. During coding, team members also made note of themes that may have been missed; analysis was concluded once new codes emerged. Once complete, the team members met to resolve any discrepancies.

## Results

### Interview data

Two prominent findings were identified: the theme of *Opportunity* and the category of *Structure & Format*. *Opportunity* refers to the frame in which the students discussed the humanities and ethics sessions. These sessions were not simply “enjoyable” or “a nice change of pace.” Rather, the students considered these sessions as a series of opportunities to gain knowledge on various topics, stop and think (i.e., engage in reflection), connect with and learn from their peers, and expand their personal growth and professional identity. As one student put it,

One thing I’ve really learned since starting this is that it’s really just taking the time to pause and maybe try to think of another, try to imagine another viewpoint. I really had a chance to stop and think about some of [the topics], and at the same time learn some techniques to, you know, stop yourself from resorting to making judgments. (M2-13)

Within this theme of *Opportunity*, six related sub-themes were identified (see Table 1 and Appendix 2 for exemplary data).

**Table 1:** Description of sub-themes

Theme: Opportunity	
Sub-theme	Description
To learn about and discuss new topics	Students discussed their excitement and appreciation of/for having the opportunity to learn about topics (e.g., prenatal screening, structural determinants of health, and advocacy) that were not only contemporary and relevant to their education and training (and future practice) but also were found to be useful in helping them explore who they want to be as doctors
To stop and think (reflection)	Students discussed the value these sessions provided them in regards almost forcing them to not rush to judgement on discussion topics, and pause, reflect, and truly consider various perspectives
To expand their own perspectives and open up to other perspectives	Although related to the “To stop and think” sub-theme, students explicitly noted how valuable they found the Humanities and Ethics sessions in opening their minds to others’ perspectives (through discussions and assigned material) and the perceived safe space to question their own thoughts and views. Similarly, students expressed the value of just meeting and connecting with other students in general through these sessions
To create balance (in education and training)	Students discussed how the sessions served as a welcomed “break” from their typical curriculum—serving as a consistent reminder that there were other ways of learning and thinking
To see whole patient	Students stated how the sessions encouraged them to cultivate their humanistic approach to medicine—that the patient is a person, and even that the patient exists within a larger social context that can influence behaviors, decisions, and opportunities
To cultivate aspects of professional identity	Students offered that the humanities and ethics sessions had advanced and expanded their understanding of what it meant to “be” a doctor, who they wanted to be as practicing physicians, and how they wanted to treat their future patients. For some students, these notions of professional identity were also tied to personal identity
Category: Structure & format	
Sub-theme	Description
Discussion-based (in small groups)	A key aspect of the sessions, from the students’ perspective, was that they were discussion based, and that these discussions were held in small groups. This structural element provided opportunities for students to hear new perspectives on various topics, practice essential communication skills, and simply engage with their peers
Topics	Students expressed the value of the topics of each session. These topics (e.g., prenatal screening, redlining, notion of deviance, health policy, among many others) yielded fruitful and engaging discussions because of their broad and multi-faceted nature. The students also found the topics relevant to their work as doctors, which further enhanced their interest and engagement
Integration (explicit and implicit)	A step beyond “Topics,” students stated the value of being able to integrate what they were learning in the sessions to what they were seeing and engaging with in the clinical setting. Whereas some students felt this was purposeful design by faculty and administration, others felt this integration was more organic
Alignment with SOM Mission	Students consistently expressed how the Humanities and Ethics sessions, from the topics raised to the discussion-based format to the outcomes of open-mindedness and enhanced humanism, were in direct alignment with the Creighton School of Medicine Mission

The analysis also identified the category of *Structure & Format* of the humanities and ethics sessions*—*the explicit aspects or features of the program that students stated had a significant effect on their perceptions of and experiences with the sessions—that is, the aspects of the sessions that were seen as facilitators and barriers to the *Opportunities*. For instance, students noted the benefits of the discussion-based format. As one student stated, “I personally enjoyed being able to discuss these topics with classmates and being able to challenge my thinking associated with each of the topics and grow from them. Because coming in I’ll have an idea, and in some instances, it changed pretty drastically through the process of being able to discuss it” (M2-45). Four sub-categories were identified in the analysis (see Table 1 and Appendix 2 for exemplary data).

Other sub-categories that were identified, but not as frequently or with enough weight to be identified as significant within the category of *Structure & Format,* were (a) *Role of Facilitators*: The faculty facilitators of the sessions were key to encouraging and sustaining discussions, as well as cultivating a safe space for students to feel open and free to share their perspective. The behavior, attitude, and approach of the facilitators clearly affected students’ experiences with the sessions; and (b) *“Fit” in Curriculum*: Students expressed relief that while the humanities and ethics sessions were “in addition to" their regularly scheduled curriculum, they did not feel it was an extra burden. They expressed that the sessions were well-built and integrated (“Fit”) into their other curricular and clinical requirements. As one student put it, “I think what stands out right now is the deliberate nature of incorporating sessions into the curriculum. It provides this opportunity to speak with your peers about some of the issues that are happening in medicine or just in the world in general” (M2-26).

### Essay data

Reference to each of the ethics and humanities learning objectives appeared in the students’ essays, but certain objectives were identified more frequently. For example, of the humanities-oriented objectives, *sociocultural components of illness & health* was identified in 99% (*n* = 41) of all students’ essays and appeared on average four times per essay*.* Both *illness & suffering* and *medicine & medical education in the context of healthcare policy and systems* were identified in 98% (*n* = 40) of all essays and appeared on average three times in each essay. Of the ethics-oriented learning objectives, *Catholic Ethical & Religions Directives (ERDs*) appeared most frequently (35%; *n* = 15) and appeared on average once per essay. The objective of developing (and reflecting on) one’s *own ethical reasoning process(es)* was identified less frequently (30%; *n* = 13) but was alluded to more often within the essays in which it did appear (three times per essay). The objective of reflecting on *clinical ethical dilemmas* was identified in 15% (*n* = 6) of the essays and only appeared on average one time per essay.

During coding, the analysis team identified a “new” learning outcome. Seeing the *whole patient* was identified in 85% (*n* = 36) of the essays and appeared, on average, about one time per essay. While this learning outcome clearly connects with the learning objectives listed above (e.g., *illness & suffering* and *sociocultural components of illness & health*), it distinctly appeared in essays. Many students explicitly stated that the humanities and ethics sessions taught them that patients are more than illnesses/ailments or corporeal beings and have emotions, experiences, and a life outside the exam room.

### End-of-year survey data

Nearly half of students (45%; *n* = 45) described their identity as changing or having changed during their first year of medical school. Additionally, 39% (*n* = 39) of students conceptualized their identity in a manner consistent with the important concepts of the university mission, such as care for the whole person (see Table 2).

**Table 2:** End-of-year survey codes and exemplars

Identity change		
Code	Exemplary data	N
Identity stayed the same or reinforced	“The core of my identity hasn't changed since starting medical school, but it has definitely given me the tools to better understand what serving others means and how to better do so” (M1-30) “I do not believe my identity as a future physician has changed much but rather, my idea of the physician I would like to be has been emphasized and fortified by the values instilled in the school's education” (M1-13)	30
Identify changed	“I used to think of my identity as a future physician as someone who would be a problem solver. I now realize that many problems our patients face cannot be readily solved in a few clinic visits. So, I think my identity as a future physician has changed to being someone who is more aware of the larger issues affecting our health and is also someone who is more humble in the face of our bodies' complexities” (M1-70) “What I have come to realize over this past year is that being a physician is a calling and shouldn't be treated as just another job by the physician themselves. Creighton has helped me to see the need to serve those who cannot serve themselves and to open my eyes to the issues around me. I hope to take this knowledge going forward, and be a physician who is compassionate with my patients but talented within whatever field I go into” (M1-60)	45
Identity still developing	“As a first-year medical student, I am still trying to identify my future as a physician. I have much to learn” (M1-40) “My identity as a future physician was molding and changing throughout my first medical school year. I do not know for sure what my identity is exactly. I know I am still trying to refine my knowledge to better care for my future patients. Right now, I am only a student and as a student, I still have a lot to learn during my second year” (M1-88)	10
No reference to identity change		15
Identity focus		
Code	Exemplar	**N**
Career choice	“I think my identity of the physician I want to be has changed in that I now relate to the importance of primary care and non-acute medical conditions. My short career as a paramedic made me only care about acute medical issues but that is changing for the better” (M1-81) “I came into medical school almost certain of the specialty I wanted to choose, but that has been shaken up due to exposure of new medical disciplines” (M1-98)	21
Mission or purpose	“My identity encompasses a desire to serve people/patients. I feel that has grown stronger at Creighton because the University fully embodies Cura Personalis and service learning. Ultimately, I want to go into a practice where I can serve the people who need it most” (M1-4) “My identity as a future physician is based on my Christian faith and the previous experiences I have had serving impoverished communities. Plain and simple, I want to serve others. Becoming a physician is the most excited, engaging way to do that” (M1-52)	39
Academic knowledge gain	“Bridging the gap between the classroom and clinic is my current focus, which has certainly been made more difficult given the current situation. For the most of my time in medical school so far, I have just had my head down pushing through classes” (M1-59) “Right now I’m just trying to learn the science and medicine, and I think being a good doctor/student doctor/resident will follow” (M1-97)	13
Personal identity dimensions	“As a future physician, I see myself relating to patients on a personal level and trying to empathize with what they are going through. Throughout my M1, I have developed more patience with different types of people and have learned to be flexible with how I talk to different people and work with them to develop a plan of care (this came especially through volunteering at Magis Clinic)” (M1-68) “I believe I have become more professional and more of a leader and developed a greater responsibility for activism” (M1-95)	21
Uncategorized		18

## Discussion

Our response to the National Academies’ call to develop rigorous program evaluation methods to assess programs that integrate the humanities, arts, and STEMM fields was effective in evaluating the integration of humanities and ethics into our newly revised pre-clerkship medical education curriculum. Particularly, it highlights the value of robust qualitative data collection and analysis to describe the outcomes of the humanities and ethics curriculum. With the documented challenge faculty have experienced in evaluating the value and outcomes of this type of curriculum (Dennhardt et al. 2016; Fins et al. 2013; Ousager and Johannessen 2010), this methodology may provide a model for other medical educators to use to evaluate their ethics and humanities programs.

Specific to the evaluated curriculum, evidence suggests that (1) students are open to engaging with humanities and ethics content during the pre-clerkship phase and that this engagement affords them an opportunity to engage in reflection and connect with their classmates (Reaction); (2) stated learning objectives for the ethics and humanities sessions are being met, and learning expands beyond the stated objectives (Learning); and (3) students’ perceptions of medicine and what it means to be a physician are expanding, suggesting personal and professional growth (Behavior).

Students’ openness to this curriculum during the pre-clerkship phase is particularly noteworthy since pre-clerkship education is characterized by high demands to learn medical science content and looming high-stakes testing. While similar humanities experiences exist in other medical school curricula—indeed, our small group design was inspired by the Humanities, Ethics, and Professionalism sessions in the Practice of Medicine course at the University of Texas Medical Branch in Galveston, and our SIS courses were inspired by the Bluebook extracurricular courses at UTHealth Houston’s McGovern Medical School—it has been encouraging to see firsthand how reflective curricular experiences help students slow down and intentionally reflect on their personal and professional development during their journey to becoming a physician. Such intentional reflection is one way for students to reconcile the implicit and explicit messages of the medical curriculum. As sociologist Fredric Hafferty points out, medical education is more than training—it is a *socialization process*, one that has the power to influence and shape students’ values and beliefs (Hafferty 2009). In other words, a student does not merely adopt new attitudes and behaviors that are “added on” to an already formed self; instead, socialization or resocialization involves some aspects of students’ selves being “*replaced* by new ways of thinking, acting, and valuing. … a dual process of moving into the new and moving away from the old” (Hafferty 2009, 63). This is a process, Hafferty claims, “for better and/or for worse—to change hearts *and* minds” (Hafferty 2009, 64). Requiring students to reflect on topics like the lived experiences of illness and suffering, implicit bias, structural inequity, and patient advocacy contributes to their personal and professional formation. It also explicitly communicates that these aspects of medicine *matter*. Encouraging critical reflection on and discussion of the accepted and presumed values of medicine and medical education may help students resist the pull of the hidden curriculum that can erode these values.

Students’ engagement appears to have achieved our desired learning outcomes; nearly all student essays referenced sociocultural components of illness and health, the lived experience of illness and suffering, and understanding medicine and medical education in the context of healthcare policy and systems. The *Opportunity* theme highlighted students’ appreciation for the opportunity to explore these topics. Students noted that the ethics and humanities sessions offered them the chance to slow down, reflect, and share thoughts with peers while offsetting the more labor-intensive learning of basic sciences. The intentionally designed, discussion-based format of the curriculum helped create an educational experience that Delese Wear and colleagues call “slow medical education”—that is, educational experiences that help students to slow down in order to offer “ways for learners to engage in thoughtful reflection, dialogue, appreciation, and human understanding with the hope that they will incorporate these practices throughout their lives as physicians” (Wear et al. 2015).

Furthermore, the design of the ethics and humanities sessions requires that students actively engage with the content rather than passively receive it in the form of lectures. This promotes deeper learning and prepares students for the human elements of medicine that are inherently variable, ambiguous, and unpredictable (Cutrer et al. 2017). This may also explain why the ethics and humanities content prompted reflection about and critical analysis of concepts beyond those stated in the learning objectives. For instance, 45% of students indicated their identity as a physician had changed during their time in the pre-clerkship years, with over one-third of the respondents conceptualizing their identity related to important university mission concepts such as service to those most in need. And 85% of student essays distinctly referenced care of the *whole patient—*recognizing that patients exist within a larger social context that can influence behaviors, decisions, and opportunities. We believe that this finding is significant, not only because of the sheer number of students who referenced the desire to care for patients as whole people but also because of the potential implications for the health systems in which students train and will eventually practice. For our students, engaging with the humanities revealed the complexities of patients’ lives, perhaps indicating the potential to apply or “translate” the humanities in ways that affect the healthcare system, including patient-clinician interactions, the distribution of healthcare resources, and patient outcomes (Chou et al. 2021).

The collective evidence across measures suggests the ethics and humanities curriculum is leading students beyond memorization of content toward more thoughtful analysis. For example, nearly one-third of students specifically referenced developing their own ethical reasoning process. Developing a new reasoning process requires critical analysis and construction of one’s own perspective of engagement with the ambiguous human elements of medicine. Also notable are the 45% of students who indicated their identity has already changed and the 10% of students who indicated that their professional identity is still developing. This suggests students’ insight into the fact that their educational experiences in medical school are inevitably shaping who they are becoming as physicians and people.

Although this research is limited to CUSOM, it provides evidence that the intentional integration of medical humanities and ethics can be evaluated. The multiple-method, qualitative approach allowed critical evaluation of students’ perceptions, learning, and behavioral intentions. Interviews demonstrated students found value in the “fit” of the content within the broader curriculum and the faculty-facilitated, discussion-based delivery, providing support for the approach of longitudinal, within-program integration. Additionally, program evaluation showed no decrease in performance in other content areas associated with the new curriculum. This reinforces the suggestion from the National Academies that a robust evaluation of curriculum content must be intentionally implemented in order to best understand the effect of this curriculum on students (National Academies of Sciences, Engineering, and Medicine 2018), particularly for schools integrating longitudinal learning.

## Limitations

All measures collected were self-reported by students as either required components of the curriculum (i.e., student essays, end-of-year survey) or through voluntary participation (i.e., student interviews) and reflect a fairly positive response to the curriculum. It is reasonable to assume some students may have responded in a socially desirable manner or simply reiterated keywords or learning objectives they heard in the course. While it is possible that student familiarity with the learning objectives had an effect on their reflection and may have been represented in the results of their essays, it was intentional to have colleagues who were external to the school and not involved in the curriculum complete the analysis of the essays and interviews to reduce the chance of bias in interpretation or analysis. Additionally, students who volunteered for the interviews may have self-selected to participate due to an interest in or enjoyment of the humanities curriculum. Despite these limitations, the triangulation of the data provides us confidence that these themes emerged across multiple measures (both required and voluntary) and moved beyond the stated learning objectives of the curriculum. Finally, these measures were all collected during the pre-clerkship curriculum, so it is not clear how these ideas might be represented or reflected once students move to the clinical phase of their education.

## Future directions

Future research should strive to also include the voices of patients as part of the program evaluation, perhaps capturing their assessment of medical students’ ability to connect, empathize, and address the lived experience of illness. The current study offers preliminary evidence that engaging in our ethics and humanities curriculum prompted students to think about their professional identity (e.g., how they intend to behave as a doctor, make clinical decisions, and select specialty choices). The end-of-year survey results suggest that a portion of students are already forecasting their future career and specialty choices, and results from student interviews suggest at least some students recognize that ethics and humanities topics are relevant to future clinical scenarios. However, behavioral intentions are not always predictive of behavior (Sheeran and Webb 2016), and further research is warranted to observe or capture students’ actual behavior (e.g., encounters with patients or standardized patients) as a result of their engagement with the ethics and humanities curriculum. Future research should follow a cohort of students from matriculation through graduation to consider whether this early introduction to ethics and humanities and the personal reflection it prompts serve as a protective factor against the demonstrated ethical erosion and decrease in empathy that occurs during the clinical years (Newton et al. 2008; Hojat et al. 2009; Neumann et al. 2011; Feudtner et al.1994; Piemonte and Kumagai 2019).

## Data Availability

The data that support the findings of this study are available from the corresponding author, CE, upon reasonable request.

## Appendix 1

Tables 3 and 4

**Table 3:** Humanities and ethics curriculum session topics

Humanities curriculum topics	Ethics curriculum topics	SIS example topics
• Introduction to Medical Humanities and Reflection • Implicit Bias and Anti-Racism • How Doctors Think: The Shortcomings of Science • Narratives and Medicine • The Nature of Suffering and the Lived Experience of Illness • The U.S. Healthcare System: How Does it Work and for Whom is it Working? • The Social Determinants of Health: Trauma as an Example • Death and Dying; Reflections on Medical Education: Who Am I Becoming? • The Patient-Physician Relationship: Physician as Provider, Partner, or Advocate? • History and Medicine: Do the Echoes of Slavery Still Haunt Contemporary Practice? • Spirituality and Meaning-Making in Medicine • The Medical Humanities in Practice: Practical Applications for Third Year	• Ethical Reasoning and Early Alzheimer’s Diagnosis • Vaccines and the Ethical and Religious Directives • Disability and Difference in Health and Disease • Prenatal Screening • Informed Consent, Privacy and Confidentiality in the Era of Big Data End-of-Life Issues in Cardiology • Ethics of Liver Transplant • Health Inequities and COVID-19 • Prenatal Diagnoses and Reproduction • Decision Making at the End of Life	• The Art of the Examination: How Observation Leads to Empathy in Healthcare • Creative Writing for Future Physicians • Is Race Real?: Racialization in Science and Medicine • A History of Disability in Medicine • The Medicalization of Deviance • Narratives of Neurodiversity • Communication Around Women’s Bodies Exploring Resistance to Vaccination: A Humanistic Perspective • Childbirth and Social Justice

## Appendix 2

**Table 4:** Description and exemplary data for sub-themes

Theme: Opportunity		
Sub-theme	Description	Exemplary data
To learn about and discuss new topics	Students discussed their excitement and appreciation of/for having the opportunity to learn about topics (e.g. prenatal screening, structural determinants of health, advocacy) that were not only contemporary and relevant to their education and training (and future practice) but also were found to be useful in helping them explore who they want to be as doctors	“It’s not necessarily listed on like what you need for Step1, which is what so many people are focused on. You’re like ‘Yea, that’s something that I kind of have an interest in and it’s cool that I can explore it.’ That it’s still part of the curriculum and helping me become a better physician. If it wasn’t a part we might not know what were’ missing out on because you’re not necessarily thinking about them so the sessions help students see topics or like understand topics that they don’t know are options” (M2-12) “Because that’s something I just didn’t really know about, and honestly, I’m not sure when I would find out about it like when I would have taken my own time to actually learn about such a thing. And I had no idea that such an issue or something like that would even impact me directly, or be really useful for me as a future physician” (M2-13) “All of our discussions have been very informative and, in my opinion, enrich our education as future physicians because they touch on components of care for other people that aren’t explicitly related to medicine” (M2-47)
To stop and think (reflection)	Students discussed the value these sessions provided them in regards almost forcing them to not rush to judgement on discussion topics, and pause, reflect, and truly consider various perspectives	“And I think from the humanities and ethics courses you learn more about critical thinking, like there’s not a simple answer. Coming from a science background you like to have a concrete answer, like this is what the answer is, this is what you do. But I’ve learned through the humanities and ethics discussions that’s not always possible” (M2-12) “So one thing I’ve really learned since starting this is that it’s really just taking the time to pause and maybe try to think of another try to imagine another viewpoint. I’ve really had a chance to stop and think about some of them [topics], and at the same time learn some techniques to, you know, stop yourself from resorting to make judgments. So I think that’s probably one of the biggest take-aways from these courses” (M2-13) “I think that with a small group and with us getting an opportunity to talk to each other, it’s kind of opened my mindset on how I go into a problem thinking like, oh, wait, there might be more to this than just automatically like going with what my gut reads first” (M2-16)
To expand their own perspectives and open up to other perspectives	Although related to “..to stop and think,” students explicitly noted the how valuable they found the Humanities and Ethics sessions in opening their mind to others’ perspectives (through discussions and assigned material), and the perceived safe-space to question their own thoughts and views. Similarly, students expressed the value in just meeting and connecting with other students in general through these sessions	“When there’s time for us to discuss I think it brings up a lot of ideas that you wouldn’t necessarily think of because you know everybody comes from somewhere different so they bring a different idea. It’s like, well, what about this like I might not think this certain way, but people could and then it’s like branching points to talk off of that” (M2-16) “People think very differently, have like very different viewpoints and whatnot. You know, we’re all med students so you’d think we all probably have like the same ideals and like values and stuff like that, but I realized quickly that that’s not always true” (M2-12) “A lot of times there’s differing opinions in the groups, which is good because you think of things like you hadn’t thought about. Especially like with a pre-reflection and I’ll go through the reading and I’ll be like, ‘Well, A, B, and C stand out to me.’ But someone else sees 1, 2, and 3, and they’re things that, like I had never thought of” (M2-16)
To create balance (in education and training)	Students discussed how the sessions served as a welcomed “break” from their typical curriculum—serving as a consistent reminder that there were other ways of learning and thinking	“It kind of helps us break things up and kind of, it helps us balance things a little bit more. It makes us realize that it’s not all about studying and it’s not all about just like getting the perfect grade or whatever the score you want on a test. It’s, there’s bigger things in life and there’s bigger things that make you a good doctor” (M2-1) “That’s what I would look forward to every week. So I think they’re a really good break from you the science-heavy science lectures and kind of recenter why you’re interested in this. At least for me—but I think for a lot of people” (M2-26) “For me, there’s something really important about taking a step back from the hard sciences. Because we work, really, really hard to master a bunch of content that has to do with science, and this gives us the opportunity to take a step back and you know really connect with the deeper reason so many of us got into medicine in the first place, which is to care for other people” (M2-47)
To see the whole patient	Students stated how the sessions encouraged them to cultivate their humanistic approach to medicine—that the patient is a person, and even that the patient exists within a larger social context that can influence behaviors, decisions, and opportunities	“I think it’s definitely helped me see a lot more of the human side of medicine. And kind of, it’s not just that you’re there and use your expertise to diagnose them and provide a treatment, but also kind of walk with them through those issues discus what the patient actually wants, to see what the patient’s family wants, and to take into account those perspectives” (M2-48) “You know, we’ve done readings about suffering, for example, which is a huge topic, but we read this one story about this man who was suffering and processing what was going on and how he was feeling and you know, for us as future physicians, we need to have, like, an awareness of what might be going on under the external façade that people put on in their day-to-day life because there’s a lot of suffering that goes unacknowledged and unnoticed by a great amount of people. And so that really stuck with me” (M2-13) “So the readings are very relevant because we about to discuss like these are human values and personal connection and how the patient is not defined by their illness, and like that we all thrive off human connection and being seen as a person” (M2-17)
To cultivate aspects of professional identity	Students offered that the humanities and ethics sessions had advanced and expanded their understanding of what it meant to “be” a doctor, who they wanted to be as practicing physicians, and how they wanted to treat their future patients. For some students, these notions of professional identity were also tied to personal identity	“There’s no doubt in my mind that it’s enhanced my experience, enhanced my education, and enhanced, you know, my capacity as a compassionate human. Yea, it’s a huge asset to our education and the School of Medicine” (M2-47) “There definitely will be ethical dilemmas that you’ll have to encounter. So I think it just kind of helps give you the tools to be that well rounded physician and try to help in those situations” (M2-29) “It changed the way I think I personally think about patients and kind of the methods of learning how to phrase things as a physician, so I know that I can get what’s best for my patient” (M2-42)
Category: Structure & Format		
Sub-theme	Description	Exemplary data
Discussion-based (in small groups)	A key aspect of the sessions, from the students’ perspective was that they were discussion based, and that these discussions were held in small groups. This structural element provided opportunities for students to hear new perspectives on various topics, practice essential communication skills, as well as simply engage with their peers	“And, you know, there’s no right and wrong. So I feel like when we’re allowed or when we’re given opportunities to discuss it amongst peers, like with the facilitator helping out, I feel like that’s when people are most receptive to it and like we learn the most from it” (M2-12) “Getting a chance to discuss it in these courses has been pretty useful because now, you know, some, some of these ideas like the liver transplant or like the redlining issues that we learned about, they still kind of still resonate in my head. And its something that I think will stay in my head from here on out and will easy to go back to as a future physician” (M2-13) “I personally enjoyed being able to discuss these topics with classmates and being able to kind of challenge my thinking associated with each of the topics and kind of grow from them because coming in I’ll have an idea and in some instances it changed pretty drastically through the process of being able to discuss it” (M2-45)
Topics	Students expressed the value of the topics of each session. These topics (e.g., prenatal screening, redlining, notion of deviance, health policy, among many others) yielded fruitful and engaging discussions because of their broad and multi-faceted nature. The students also found the topics relevant to their work as doctors which further enhanced their interest and engagement	“These topics are not cut and dry, there is no right or wrong answer and one of the greatest things you can learn is from the experience of others, you know, broadened my horizons and opened my eyes to a lot of things that could be going on with patients that we, at least I, personally didn’t really think about or know about before” (M2-47) “I thought that the topics were very relevant and educational. I learned quite a bit that I was not aware of before and how that interplays with medicine” (M2-17) “It’s given me a chance to have meaningful conversation with a lot to classmates that I wouldn’t normally have gotten to and especially on topics, you know, not necessarily directly related to like science” (M2-27)
Integration (explicit and implicit)	A step beyond “Topics,” students stated the value of being able to integrate what they were learning in the sessions to what they were seeing and engaging with in the clinical setting. Whereas some students felt this was purposeful design by faculty and administration, others felt this integration was more organic	“I can’t remember if that was during our repo block or during our endo block, all the dates are kind of merged in my head, but I think that it was really relevant to how we might have to counsel a potential mother in future practice, even if we don’t go into OB-GYN, but like even if we are in like primary care, or honestly any other specialty. You might have to come across the discussion, similar to that one” (M2-17) “I think what stands out right now is the deliberate nature of incorporating sessions into the curriculum. It provides this opportunity to speak with your peers about some of the issues that are happening in medicine or just in the world in general” (M2-26) “It’s the balance. I mean, it’s good to have it and it’s good to have it incorporated. Even though it can be a bit of a, you know, sometimes I’m like it’s still a good thing. Like, you got to eat your vegetables, you know?” (M2-42)
Alignment with SOM Mission	Students consistently expressed how the Humanities and Ethics sessions, from the topics raised to the discussion-based format to the outcomes of open-mindedness and enhanced humanism, were in direct alignment with the Creighton School of Medicine Mission	“I think in so many ways this course ties into the kind of Creighton mentality—it ties into, you know, the goals and desires of myself and a lot of my classmates and without it I think it would just feel foreign, it’s been hard to connect what we’re studying to what we’re actually going to be doing” (M2-47) “The reason I picked Creighton was that it’s focused on treating the whole individual as opposed to some other med schools where it’s like, ‘Here’s the science, here’s the medicine.’ you know, hand them out. If these courses weren’t here Creighton wouldn’t be following its mission. It wouldn’t be living out, so to speak, that mission statement and there’d be that gap. What other school do you get this, you know? Not many” (M2-43) “The sessions get you out of that framework of thinking of things as technicalities and as a scientific question and approaching the human side of medicine that I think the Creighton mission statement really tries to emphasize” (M2-48)
